# Results and current approach for Brachial Plexus reconstruction

**DOI:** 10.1186/1749-7221-6-2

**Published:** 2011-06-16

**Authors:** Jayme A Bertelli, Marcos F Ghizoni

**Affiliations:** 1 Center of Biological and Health Sciences, University of Southern Santa Catarina (Unisul). Tubarão, SC, Brazil; 2 Center of Biological and Health Sciences, University of Southern Santa Catarina (Unisul). Tubarão, SC, Brazil

## Abstract

We review our experience treating 335 adult patients with supraclavicular brachial plexus injuries over a 7-year period at the University of Southern Santa Catarina, in Brazil. Patients were categorized into 8 groups, according to functional deficits and roots injured: C5-C6, C5-C7, C5-C8 (T1 Hand), C5-T1 (T2 Hand), C8-T1, C7-T1, C6-T1, and total palsy. To restore function, nerve grafts, nerve transfers, and tendon and muscle transfers were employed. Patients with either upper- or lower-type partial injuries experienced considerable functional return. In total palsies, if a root was available for grafting, 90% of patients had elbow flexion restored, whereas this rate dropped to 50% if no roots were grafted and only nerve transfers performed. Pain resolution should be the first priority, and root exploration and grafting helped to decrease or eliminate pain complaints within a short time of surgery.

## Introduction

Brachial plexus lesions are a tragic condition that usually affects young adults, with significant socioeconomic implications. Only four decades ago, brachial plexus surgery still was approached with considerable pessimism. As recently as the 1996 International Society for Orthopaedic Surgery and Traumatology (SICOT) in Paris, it was concluded that surgical repair of these lesions was almost impossible and, even when performed, did not guarantee a useful result [1]. However, the ongoing increase in the number of civilian brachial plexus lesions due to motorcycle accidents has, without a doubt, promoted interest in this field, and recent years have witnessed tremendous progress in surgical techniques for brachial plexus repair. At our institution, between January 2002 and December 2008, 335 patients suffering from supraclavicular brachial plexus palsy underwent surgical repair. In the present report, we review our results and current approach to treatment. Written informed consent were obtained from patients for publication of clinical cases and accompanying images. In advance of any data collection, the protocol of the present study was approved by the local ethics committee. All patients provided their written informed consent prior to their participation, in accordance with the Declaration of Helsinki guiding biomedical research involving human subjects.

### Diagnosing brachial plexus palsies

In half of our patients, electrophysiological studies were available preoperatively. In 102 patients, magnetic resonance imaging (MRI) of the brachial plexus, including the spinal cord, was obtained, whereas computed tomomyelography (CT myelography) was performed in all cases. The clinical diagnosis of root involvement was correct in 85% of our patients. Extremely reliable tests or signs were a supraclavicular Tinel's sign to indicate a graft-able root, and a Horner's sign to indicate lower root avulsion [2]. Electrophysiological studies did not contribute, in any way, to identifying indications for surgery or to surgical planning. Consequently, we no longer request electrophysiological studies preoperatively. MRI was useful merely to document avulsion of the lower roots. However, Horner's sign was 96% predictive of lower root avulsion [2]. MRI was not helpful in identifying a graft-able root at the C5 or C6 level, because of poor visualization of the intradural portion of these roots. This is the main reason for interest in CT myelography: confirming that a root stump located in the supraclavicular region during dissection is in continuity with the spinal cord and, thereby, eligible for grafting.

### Timing of surgery

Patients with total palsy of the brachial plexus following a traffic accident have almost no chance of spontaneous recovery. In our series of patients with total palsy, spontaneous recovery was not observed. This is different for partial injuries, in which some spontaneous improvement might occur. We prefer to operate on such patients after the third month but before the sixth month after injury. Some of our patients were operated upon after the seventh month with good results. However, more than 9 months after trauma, our results declined dramatically.

In partial injuries, we observed some good results even 10 months after surgery when distal nerve transfers were employed. This was not observed in patients with total palsy in whom nerve grafts were used 10 months or more following the accident [3].

### Definition of paralysis according to root involvement

#### C5-C6 root injury (n = 54)

**Figure 1:**
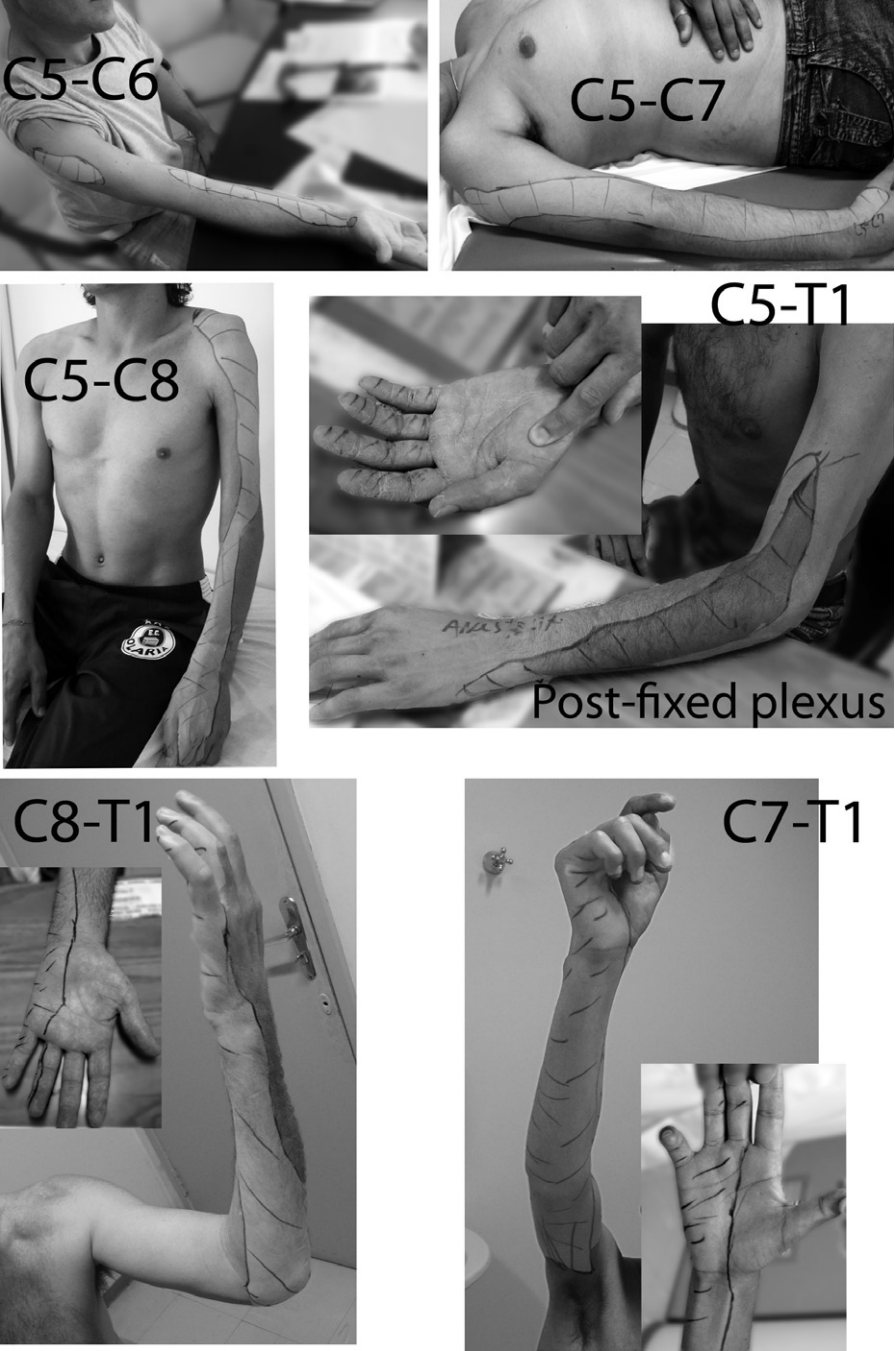
**Zones of lost protective sensation with the different types of brachial plexus palsy**. Mapping was performed following assessments using Semmes-Weinstein monofilaments. In those with a C5-C6 injury, hand sensation was totally preserved. In the C5-C7 injury group, hand sensation was decreased, but still was within the normal range. A longitudinal area of absent protective sensation was present on the lateral aspect of the forearm and arm. In the C5-C8 palsy group, there was a similar longitudinal area along the lateral side of the arm and forearm, associated with no protective sensation. The dorsal side of the hand also was now markedly affected. On the palmar aspect of the hand, sensation decreased to a variable degree. Almost half of the patients had normal sensation, and the remaining half experienced thumb anesthesia. In those with a C5-T1 lesion with post-fixation of the plexus, only a small zone was observed in which there was preserved sensation over the medial side of the forearm. Hand sensation was markedly reduced. The thumb was anesthetized, but protective sensation was demonstrated in the long fingers (inset). In the C8-T1 palsy group, a loss of protective sensation was evident on the medial side of the forearm and in the ulnar fingers. In the C7-T1 injury group, the inner aspect of the arm also was affected, together with additional involvement of the long finger.

This group consisted of those patients with palsy involving shoulder abduction and external rotation, elbow flexion, and forearm supination. The coracobrachialis remained innervated in all patients. The flexor carpi radialis and pronator teres functioned, but were weak. Wrist flexion was largely preserved, because the palmaris longus and the flexor carpi ulnaris were unaffected. The upper head of the pectoralis major was paralyzed or weak; but, during resisted adduction, there was no apparent atrophy of the muscle. Hand grasp strength was 68% that of the normal side, and pinch strength was 80% of normal. Wrist extension strength was 55% that of the normal side, while elbow extension was 66% the normal value (Table 1). The hand exhibited normal sensation. One zone of lost protective sensation was observed along the lateral aspect of the forearm extending towards but not necessarily reaching the thumb. A second zone was noted over the deltoid chevron (Figure 1).

**Table 1:** Grasping, pinch and wrist and elbow extension strength in the different group of palsies.

Type of palsy	*Grasping (Kg)*	*Pinch (Kg)*	*Wrist extension (Kg)*	*Elbow extension (Kg)*
**C5-C6**	26.3(95% CI, 22.9-29.7)	8 (95% CI, 7.1-8.8)	7.1(95% CI, 6.1-8.1)	7.8 (95% CI, 6-9.5)

**C5-C7**	14.7(95% CI, 8.7-20.7)	6(95% CI, 4.8-7.1)	5.6 (95% CI, 5.1-6.2)	4.8(95% CI, 4.2-5.4)

**C5-C8**	13(95% CI 9.7-16.3)	4(95% CI 2.8-5.3)	paralyzed or extremely weak	paralyzed

**C5-T1 (postfixed)**	extremely weak	extremely weak	paralyzed or extremely weak	paralyzed

**Normal**	38(95% CI, 36.6-39.4	10.1(95% CI, 9.2-10.9)	13(95% CI, 11.4-14.5)	11.8 (95% CI, 10.4-13.2)

Strength with grasping, pinching and wrist and elbow extension in the different palsy groups and in the normal contralateral limb - In all groups, strength was significantly decreased relative to the normal side (p < 0.005). Grasp strengths were 70%, 40% and 36% that of the normal limb in those with C5-C6, C5-C7 and C5-C8 injuries, respectively. All inter-group differences were statistically significant; however, only the difference between the C5-C6 and C5-C7 groups can be considered clinically relevant. Pinch strengths were 80%, 60% and 40% of normal with C5-C6, C5-C7 and C5-C8 lesions, respectively; these differences all were significant, both statistically (p < 0.05) and clinically. Wrist extension strengths were 55%, 43% and 0% in the C5-C6, C5-C7 and C5-C8 palsy groups, respectively; and corresponding elbow extension strengths were 66%, 41% and 0%. These differences in wrist and elbow extension strength all were statistically significant (p < 0.05).

#### C5-C7 root injury (n = 18)

The clinical picture with this lesion was similar to what we observed in the C5-C6 palsy group. Wrist, finger and elbow extension were preserved, but there tended to be a greater loss of strength than in the C5-C6 patients (Table 1). The upper portion of the pectoralis was atrophied. The latissimus dorsi was paralyzed in half of the patients. The flexor carpi radialis and pronator teres were paralyzed. There was an extended sensory deficit, but no loss of protective sensation in the fingers. Hand grasp strength was just 39% that observed in the normal contralateral limb, whereas pinch strength was 60% the normal value. Wrist extension and triceps strength were 43% and 41% that of the normal side, respectively. Relative to the C5-C6 group, grasp, pinch, triceps and wrist extension strength were significantly decreased. There was a reduction in sensation in all fingers, but especially in the thumb. However, protective sensation was preserved throughout the entire hand. There was a continuous longitudinal area of anesthesia along the lateral aspect of the forearm and arm, and over the deltoid chevron (Figure 1).

#### C5-C8 root injury (T1 Hand, n = 63)

In addition to the shoulder and elbow flexion palsy, the teres major, latissimus dorsi and triceps all were paralyzed. The pectoralis major was paralyzed. Wrist extension was paralyzed in all patients. However, some patients could extend their wrist with the help of thumb and finger extensors. In these cases, wrist extension strength did not exceed 1 kg. In a few patients, only extension of the thumb and index finger was preserved. The triceps, flexor carpi radialis, pronator teres and flexor carpi ulnaris were paralyzed. Wrist flexion was possible, thanks to the palmaris longus. Pronation was possible because the pronator quadratus functioned. Hand grasp strength was 36% that of the normal side, and pinch strength 40% of normal (Table 1). In comparison with the C5-C6 ± C7 groups, grasping and pinch strength were significantly reduced. There was a continuous longitudinal zone of lost protective sensation over the lateral forearm, lower arm and deltoid chevron, as in the previous group. However, this region was wider, affecting 1/3 of the limb circumference. Contrary to the previous group, the dorsal aspect of the hand, including the dorsal ulnar side, now was affected. With respect to the fingers, there was no particular pattern of sensory disturbance. For instance, a few patients presented with thumb anesthesia, but others exhibited normal sensation in all fingers. In no instance was complete anesthesia of all fingers observed (Figure 1). Horner's sign was absent.

#### C5-T1 root injury with partially preserved finger flexion and Horner's sign (T2 Hand, n = 12)

In these patients, the shoulder was completely paralyzed. Adduction was impossible, because the pectoralis major was totally paralyzed in all patients. Finger and wrist extension were paralyzed. Wrist flexion was weak, but preserved in half of the patients because the palmaris longus remained functional. Finger flexion was noted in all cases, but it was incomplete in excursion, and in no hand were all fingers functional. Grasping and pinch strength were not measurable due to extreme weakness. Thumb anesthesia was frequent. The dorsal aspect of the hand was completely anesthetized. The lateral longitudinal bundle, over the forearm and arm, was wider, now comprising 2/3 of the limb circumference (Figure 1). There was a zone of normal sensation over the ulnar border. It is our impression that these patients had either an undetected partial root injury of T1, though CT tomomyeloscans confirmed avulsion; or, more likely, post-fixation of the brachial plexus by T2. We now call this cohort of patients the T2 hand group.

#### C8-T1 root injury (n = 9)

Shoulder and elbow flexion were normal (Table 2). Wrist and finger extension were preserved. The flexor carpi radialis was preserved, but the flexor carpi ulnaris and palmaris longus were paralyzed. The pronator teres was preserved. Intrinsic muscles of the hand were partially preserved. The flexor pollicis longus was paralyzed. Strength of wrist extension was 73% that of the normal wrist. Elbow flexion was equal to the contralateral side. Sensory disturbances compromised the ulnar aspect of the hand and forearm. Horner's sign was present in all cases, and CT myeloscans indicated avulsion of both C8 and T1 in all patients.

**Table 2:** Values of strength for elbow flexion/extension and wrist extension in C8-T1 and C7-T1 palsies.

Type of palsy	*Elbow Flexion/Kg*	*Elbow Extension/Kg*	*Wrist Extension/Kg*
**C8-T1**	16.2(95% CI, 13.5-18.9)	12.5 (95% CI, 7.9 17)	9.5(95% CI, 6.7-12.2)

**C7-T1**	11.6 (95% CI, 10.6-12.6)	3(95% CI,1.7-4.2)	6(95% CI, 3.6-8.3)

**Normal**	19(17.2-20.7)	11.8 (10.4-13.2)	13(95% CI, 11.4-14.5)

Values of strength for elbow flexion/extension and wrist extension in C8-T1 and C7-T1 palsies. Strength in C7-T1 injuries was significantly (p < 0.05) reduced relative to the normal, contralateral limb. The rates for the C8-T1 injury group did not differ from normal values. In the C7-T1 group, elbow and wrist extension strengths were 25% and 46% those of the normal side, respectively.

#### C7-T1 root injury (n = 22)

These patients with more extensive palsy recovered spontaneously in the territory of the upper roots of the brachial plexus. Shoulder and elbow range of motion were normal. However, strength was markedly reduced relative to the patient's normal side, and relative to the affected limb in the C8-T1 palsy group (Table 2). For instance, elbow flexion strength was 61% and triceps strength just 25% that of the normal side. Wrist extension was weak, corresponding to 46% that of the normal side. When patients extended their wrist, radial deviation occurred. Both the extensor carpi radialis brevis and longus functioned, but the extensor carpi ulnaris was paralyzed. Sensory disturbance was present along the ulnar aspect of the hand, forearm and arm. As opposed to the C8-T1 group, decreased sensation also was apparent in the third finger. Thumb sensation was normal.

#### C6-T1 root injury (n = 4)

We observed 4 patients with paralysis of finger flexion/extension, accompanied by weak wrist extensors and triceps paralysis. Triceps paralysis and weak wrist extension were the main differences in this versus the previous group. Shoulder motion and elbow flexion were almost normal. Surgery was not performed for brachial plexus exploration; hence, the status of the roots was not inspected directly. On CT myeloscan, avulsion of C7-T1 was confirmed in all cases. The nature of the lesion affecting C6 was not clear. Most likely, there was partial injury with spontaneous recovery. Of importance is that, in this group of patients, elbow extension reconstruction is mandatory.

#### Total Palsy (n = 168)

In these patients, the clinical picture was a flail limb. All patients presented with a Horner's sign. The sensory deficit included the entire limb, except for the inner aspect of the arm.

### Surgical Treatment

#### Upper Type Palsies (C5-C6 ± C7)

**Figure 2:**
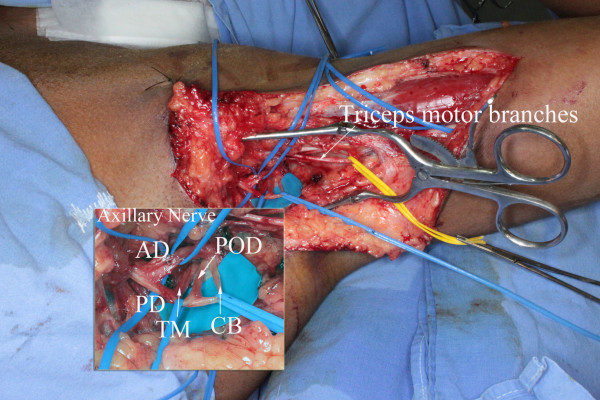
**Intra-operative view of a left axillary approach to neurotize the anterior division (AD) of the axillary nerve and the teres minor motor branch**. Through this same approach, ulnar nerve fascicles are concomitantly transferred to the biceps motor branch. (PD) posterior division of the axillary nerve and its branches: (TM) teres minor motor branch, (POD) branch to the posterior deltoid muscle, and (CB), the upper arm lateral cutaneous nerve.

In these patients, elbow flexion and shoulder abduction/external rotation are the missing functions that require reconstruction. When roots are avulsed, which occurred in only 22% of our patients, reconstruction is achieved by triple neurotization: (1) the accessory nerve is transferred to the suprascapular nerve; (2) the triceps long head motor branch is transferred to the anterior division of the axillary nerve and to the teres minor motor branch; and (3) fascicles of the ulnar nerve are transferred to the biceps motor branch [4]. We now perform triceps-to-axillary nerve transfers using an axillary approach (Figure 2). Comparing results for patients with root avulsions treated by triple neurotization exclusively versus those who had roots grafted to the upper trunk plus triple neurotization, we observed better results for the combined root grafting plus nerve transfer procedure [5]. We believe that root grafting was helpful for reinnervation of antagonist muscles or shoulder stabilizers, which were not addressed by the nerve transfer intervention. For instance: when elbow flexion is resisted, pectoralis major contraction is easily perceived, even though the pectoralis major is not an elbow flexor. The pectoralis major contracts to stabilize the shoulder joint. Also, we observed that patients with C5 and C6 root grafting in whom the suprascapular nerve was transferred to the XI^th ^cranial nerve and fascicles of the ulnar nerve were transferred to the biceps motor nerve, but who had no triceps transfers to the axillary nerve, exhibited less external rotation recovery than patients who underwent triple nerve transfers. In this group of patients, external rotation is the most difficult motion to restore. Therefore, contrary to what other surgeons have proposed,[6] we believe that not only the anterior branch of the axillary nerve, but also the teres minor motor branch should be consistently neurotized by triceps nerve branches. Hence, it is our policy now that, even when we have two graft-able roots (C5+C6), we graft the roots to the upper trunk and perform a triple nerve transfer.

Our overall results for reconstruction of upper-type lesions of the brachial plexus are encouraging. Both full abduction and full external rotation of the shoulder were restored in 15% among those in the C5 and C6 nerve root avulsion group reconstructed by triple nerve transfer, in 67% of the patients who received C5 nerve root grafting plus a triple nerve transfer, and in 33% of patients who received a C5+C6 nerve root graft group, plus transfer of cranial nerve XI to the suprascapular nerve and ulnar nerve fascicles to the biceps motor branch. The average percentages of elbow flexion strength recovery, relative to the normal, contralateral side, were 27%, 43% and 59% for the C5-C6 nerve root avulsion, C5 nerve root graft, and C5+C6 nerve root graft groups, respectively. Hence, it seems that combining grafted roots with distal nerve transfers also improves elbow flexion strength.

#### T1 Hand

When the C5 root was available for grafting, it was grafted either to the anterior or posterior division of the upper trunk. To date, we have not observed clear differences in recovery of motion attaching nerve grafts to the anterior versus posterior division of the upper trunk, but this may be because of the few patients like this that we have had. In the 'T1 hand' group of patients, elbow extension has required reconstruction. In a few patients, we have used the median nerve to neurotize the biceps motor branch, while ulnar nerve fascicles were used to neurotize the triceps long head motor branch. None of these patients recovered satisfactory elbow flexion/extension. Consequently, we have abandoned the procedure of triceps reinnervation by ulnar nerve fascicles.

In another group of 4 patients, we tried intercostal nerve transfers to the triceps long head motor branch, with fruitless results, either because of poor reinnervation or poor motor control. Our current approach is to transfer the levator scapulae motor branch to the triceps long head motor branch, aided by a sural nerve graft. Recovery of elbow extension is not strong, but is fully under voluntary control, and control over elbow flexion-extension is important to stabilize the elbow when tendon transfers are needed for thumb and finger extension reconstruction. In this regard, we should highlight an important point. If the motor fascicles of the flexor carpi ulnaris (FCU) are transferred for elbow flexion through biceps reinnervation, and then the FCU is transferred to the extensor digitorum communis (EDC) and extensor pollicis longus (EPL) for reconstruction of a radial nerve palsy, results will be very poor. When the patient extends the thumb and fingers by activating the transferred FCU, the elbow flexes concomitantly. In the T1 hand, ulnar nerve fascicles to the intrinsic muscles of the hand should be used for finger flexors instead of fascicles for the FCU, as originally proposed by Oberlin et al.[7] Transferring fascicles of the ulnar nerve to the intrinsic muscles of the hand does not downgrade hand fucntion [5].

**Figure 3:**
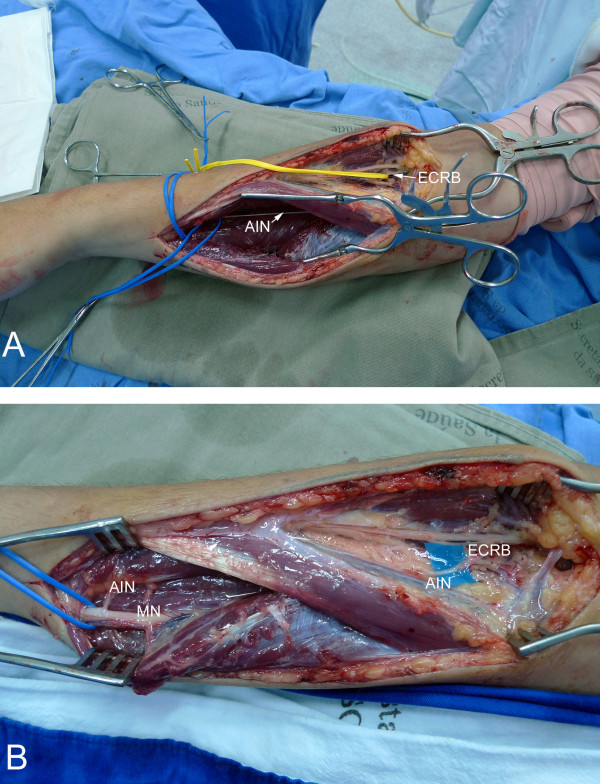
**A) Intra-operative view of transferring the pronator quadratus motor branch (i.e. anterior interosseous nerve) to the extensor carpi radialis motor branch (ECRB)**. B) After sectioning, the proximal stump of the anterior interosseous nerve (AIN) was flipped proximally for suturing to the extensor carpi radialis brevis motor branch, which was dissected and sectioned proximally, and flipped distally. The distal stump of the anterior interosseous nerve was sutured to a motor fascicle of the median nerve, (MN) end-to-end, to restore pronation.

In patients with lesions extending from C5-C8, wrist extensors are always paralyzed. However, in half of the patients, finger and thumb extensors are working and wrist extension can be accomplished by the activation of the extensor digitorum communis and the extensor pollicis longus. In one quarter of these patients, thumb and finger extension and all wrist flexors, excepting the palmaris longus, are paralyzed. In these patients, the flexor carpi ulnaris is not available for thumb and finger extension reconstruction. The pronator teres is paralyzed, but the pronator quadratus is functioning. Hence, in these dramatic cases, we have successfully transferred the motor branch of the pronator quadratus (i.e. anterior interosseous nerve) to the motor branch of the extensor carpi radialis brevis. After sectioning the anterior interosseous nerve, the proximal stump was turned proximally and sutured to the extensor carpi radialis brevis motor branch, while the distal branch, was connected to one motor fascicle of the median nerve to the thumb intrinsic muscles (Figure 3). Twelve months after surgery, all our 4 patients could raise their wrist against gravity and pronate actively their forearm. Thumb and finger extension were achieved by a tenodesis effect. There was no deleterious functioning of the flexor pollicis longus and of the flexor digitorum profundus to the index finger because the major branches to these muscles emerged very proximally and could be preserved during the dissection of the anterior interosseous nerve.

Another interesting observation we have made is that, in general in the T1 hand, intra-operative electrical stimulation of the median nerve produces stronger finger motion than stimulation of the ulnar nerve. Hence, in the T1 hand, we always dissect both the ulnar and median nerves, stimulate them with a nerve locator, and then use the 'stronger' nerve as a donor of fascicles for biceps reinnervation.

#### Total Palsy

##### A) With graft-able roots

In 87% of our patients with total palsy, a graft-able root was available.^2 ^In these patients, the accessory nerve is always transferred to the suprascapular nerve, which yields an average of 57° of shoulder abduction. When two roots were available for grafting, typically C5+C6, C5 was grafted to the anterior division of the upper trunk, and C6 was grafted to the posterior division. Sural grafts were less than 10 cm long. After surgery, forearm muscle reinnervation was not useful. Some patients recovered wrist flexion and some finger flexion, albeit weak. We consider M3-level finger flexion useless, because our patients did not use their hand for active grasping. Elbow flexion/extension could not be restored simultaneously. Elbow flexion was M3 or more in 85% of these patients. Elbow flexion always was accompanied by pectoralis major contraction [8].

In a second group of patients, the C5 root was grafted more distally, either to the lateral cord or to the musculocutaneous nerve. When available, the C6 root was grafted to the radial nerve. The pectoralis major was reinnervated by branches to the platysma, whereas the triceps long head was reinnervated by branches to the levator scapulae nerve. Elbow flexion scoring M3 or better was identified in 91% of patients, a score slightly better than when short grafts were connected to the upper trunk [8,9]. These findings might result from double lesions of the musculocutaneous nerve, which occurred in 18% of our cases [9] and would have been passed undetected when only the supraclavicular region of the brachial plexus was explored[8]. Transferring the levator scapulae motor branch to the triceps long head restored elbow extension predictably, albeit weakly. Using the C6 root to reconstruct the radial nerve largely was unpredictable, both with respect to elbow extension and wrist and finger extension. Though partially successful, definitive conclusions regarding the transfer of the platysma motor nerve to the medial pectoralis nerve cannot be drawn.

In a third group of patients, we grafted the C5 root to the lateral cord using a vascularized ulnar nerve graft[10]. Results were poor. In our opinion, the reasons for failure are related to the length of our grafts allied with the unfavorable internal morphology of a trunk graft. It is possible that regeneration in long, vascularized trunk grafts is worse than what occurs after the same repair using a sural nerve graft. We have since abandoned the use of vascularized ulnar nerve grafts. In total palsies, our current trend is to graft roots and donor nerves directly to recipient nerves using longer grafts.

In total palsies of the brachial plexus, it is imperative to graft viable roots. This offers not only a good potential for recovery, but also treats brachial plexus pain. In total lesions, 84% of the patients suffered from pain and almost 84% have a graft-able root[2,11]. Pain subsided in half of these patients in the days after grafting. We have postulated that pain in brachial plexus injuries stems from ruptured rather than avulsed roots,[12] challenging current concepts which blame deafferentation as the origin of pain[13]. In patients who have been grafted but pain persists, we have attributed pain to the growth of axons, because this process is associated with the large production of neurotrophic factors that produce pain[14,15].

##### B) Without graft-able roots

In these patients, not only are there no roots available for grafting, but donor nerves for transfer - like the accessory and phrenic nerve - may not be available either. After surgery, only half of these patients achieved recovery of elbow flexion when we used the phrenic nerve, contralateral C7 or hypoglossal nerve. For suprascapular nerve neurotization, we used the accessory nerve, contralateral C7, hypoglossal nerve, cervical plexus and platysma motor branch. Shoulder abduction was restored in half the patients, to an average of 28°. Results for reconstruction of total palsies without a graft-able root clearly were worse than when a root was eligible for grafting. This suggests that root grafting is better than extraplexual nerve transfers for elbow flexion reconstruction. Extension of the trauma also may have affected donor nerves for transfer. For instance, results for shoulder abduction following cranial nerve XI to suprascapular nerve transfers were poor, relative to when a root was available for grafting. This might reflect not only an associated lesion of the accessory nerve, but also an extended lesion affecting the suprascapular nerve.

#### Lower Type Palsies

In all patients, thumb and finger flexion was reconstructed by transferring the brachialis muscle to the flexor digitorum profundus and flexor pollicis longus[16]. Tension was adjusted according to each patient's needs. Restoration of at least 2 kg of grasping strength allowed our patients to use their hand during daily activities. Some patients recovered up to 8 kg of strength, because more tension was applied during the transfer. This augments the power of grasping when the elbow is extended as a consequence of a tenodesis effect. In addition, strong wrist extension is helpful to increase the range of motion and power of finger flexion. However, when more tension is applied for the brachialis transfer, hand span may be jeopardized. It is particularly important to reconstruct thumb motion, more than that of the fingers. In two patients, besides a brachialis transfer, during a second surgery we transferred the brachioradialis to the flexor digitorum superficialis and flexor pollicis longus. These two patients experienced a 50% improvement in grasp strength, with preserved hand span.

When paralyzed, elbow extension was reconstructed by transferring the posterior deltoid to the triceps using a fascia lata graft. All 4 patients recovered enough stability of the elbow to allow us to proceed to tendon transfers for hand reconstruction. Only one patient recovered M4 elbow extension, with the remaining scoring M3-. If surgery is performed within 6 months of injury, we now prefer to reconstruct elbow extension by transferring either the motor branch of the posterior deltoid or the motor branch of the teres minor, as we have done for tetraplegics[17,18].

Finger extension, when absent, was successfully reconstructed by transferring the supinator motor branches to the posterior interosseous nerve[19,20]. Tenodesis of the EDC and EPL produced poor results. Poor outcomes also resulted from transferring the extensor carpi radialis longus or brachioradialis to the EDC.

Thumb stabilization by tenodesis of the abductor pollicis longus on the dorsal side of the radius or to the FCU produced limited improvement. In contrast, excellent results for thumb stabilization and spanning occurred after transferring the supinator motor branch to the posterior interosseous nerve[20]. Good results also were observed in two patients with chronic lesions who had their supinator muscle transferred to the extensor pollicis brevis, aided by a tendon graft [21]. In addition, in 3 patients with longstanding lesions, thumb and finger extension were successfully reconstructed by transferring a free gracilis muscle reinnervated by the supinator motor branch. Free muscle transfer is our preferred method of reconstruction of thumb and finger extension in patients with lower type palsy of the brachial plexus lasting for more than 12 months. When needed, stabilization of the thumb interphalangeal joint was achieved by transferring half of the flexor pollicis longus to the extensor pollicis longus[22].

**Figure 4:**
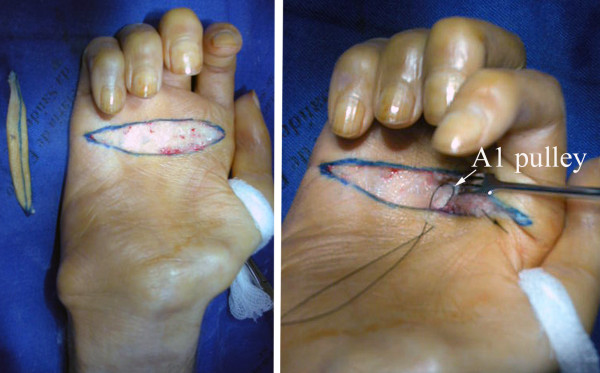
**Intra-operative view of a pulley dermodesis for correction of metacarpophalangeal hyperextension in a patient with a lower type palsy of the right brachial plexus**. After resection of a cutaneous ellipse centered on the distal palmar crease, the A1 pulley was sutured to the palmar aponeurosis and proximal dermis.

Intrinsic muscle function reconstruction was attempted by removing an ellipse of skin over the distal palmar crease and suturing the proximal dermis and palmar aponeurosis to the A1 pulley (Figure 4). If good function of the extrinsic extensors of the fingers was preserved, or reconstructed by nerve transfers, good results were observed. Otherwise, the results were poor. In patients with poor results, we have tried to improve intrinsic function by transferring the extensor carpi radialis brevis, prolonged by four-tailed tendon grafts, to the interosseous tendon, as proposed by Brand[23]. There was no improvement in proximal interphalangeal joint extension. When the extensor indicis proprius was preserved, it was successfully transferred to reconstruct thumb abduction.

**Figure 5:**
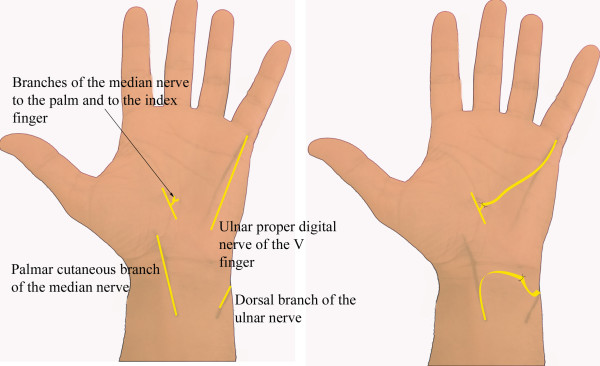
**Schematic representation of procedures to restore sensation on the ulnar side of the hand in patients with a lower-type palsy of the brachial plexus**. Either the palmar cutaneous branch of the median nerve was transferred to the dorsal branch of the ulnar nerve, or the proper digital nerve of the little finger was sutured to fascicles of the median nerve to the palm, either in association or not in association with fascicles raised from the proper ulnar digital nerve of the index finger.

Sensation on the ulnar side of the hand was reconstructed either by transferring the palmar branch of the median nerve to the dorsal branch of the ulnar nerve, or by connecting the proper digital ulnar nerve of the little finger with fascicles of the median nerve to the palm or index finger (Figure 5). We now prefer to reconstruct protective sensation using the proper digital nerve of the little finger, because when the dorsal branch of the ulnar nerve was reinnervated, sensation was not restored on the ulnar side of the little finger, only on the ulnar side of the hand.

## Conclusions

In partial injuries, brachial plexus surgery is highly rewarding. In total palsies, motion of the shoulder and elbow can be predictably reconstructed, provided that a root is available for grafting. If no root is available, only half of the patients will experience improved motion. Useful reconstruction of hand function is not yet possible with total lesions. Finger flexion or wrist extension scoring M3, although reconstructed in a few cases, was not much appreciated by our patients. Thoracobrachial and forearm abdominal grasping was their preferred method for holding objects. Treatment of pain should be a first priority. In this regard, roots should be explored and grafted.

## Competing interests

The authors declare that they have no competing interest.

## Authors' contributions

Jayme A. Bertelli MD, PhD and Marcos F. Ghizoni, MD performed surgery, patient evaluations and manuscript redaction. All authors read and approved the final manuscript.
